# Oscillations for Neutral Functional Differential Equations

**DOI:** 10.1155/2014/124310

**Published:** 2014-08-18

**Authors:** Fatima N. Ahmed, Rokiah R. Ahmad, Ummul K. S. Din, Mohd S. M. Noorani

**Affiliations:** School of Mathematical Sciences, Faculty of Science and Technology, Universiti Kebangsaan Malaysia, Malaysia

## Abstract

We will consider a class of neutral functional differential equations. Some infinite integral conditions for the oscillation of all solutions are derived. Our results extend and improve some of the previous results in the literature.

## 1. Introduction 

During the past few decades, neutral differential equations have been studied extensively and the oscillatory theory for these equations is well developed; see [1–19] and the references cited therein. In fact, the developments of oscillation theory for the neutral differential equations began in 1986 with the appearance of the paper of Ladas and Sficas [15]. A survey of the most significant efforts in this theory can be found in the excellent monographs of Győri and Ladas [12] and Agarwal et al. [1].

Consider the first-order neutral differential equations of the form

(1) $$\begin{array}{c} {\left[r\left(t\right)\left(x\left(t\right)+px\left(t-\tau \right)\right)\right]}^{\prime }+q\left(t\right)x\left(t-\sigma \right)=0,\;\;t\geq t_{0}, \end{array}$$

where

(2) r,q∈C[[t0,∞),(0,∞)],    p∈R, τ∈(0,∞), σ∈R+.

There are numerous numbers of oscillation criteria obtained for oscillation of all solutions of (1). In particular, many various sufficient conditions for oscillation are established in [3–5, 9–15, 18, 19]. In reviewing the literature, (1) is much studied in the case when

(3) $$\begin{array}{c} \overset{\infty }{\underset{t_{0}}{\int }}q\left(t\right)dt=\infty , \end{array}$$

which has been considered as an essential condition for the oscillation.

However, Yu et al. [19] considered (1) when *r*(*t*) ≡ 1, *p* = −1 in the case when (3) does not hold (in this case (1) is said to have integrally small coefficients).

In [9], Gopalsamy et al. studied (1) when *r*(*t*) ≡ 1, −1 ≤ *p* ≤ 0 and proved that every solution of (1) is oscillatory if

(4) $$\begin{array}{c} \underset{t\to \infty }{\lim}\;\inf\overset{t}{\underset{t-\sigma }{\int }}q\left(s\right)ds>1+p. \end{array}$$

In [5], some finite integral conditions for oscillation of all solutions of (1) when *r*(*t*) ≡ 1 are given under less restrictive hypothesis on *p*. See also Grammatikopoulos et al. [10], Ladas and Sficas [15], and Al-Amri [4].

Recently, Ahmed et al. [2, 3] investigated the oscillation behaviour of (1) and obtained some new oscillation results. Additional results on the oscillation behaviour of (1) can also be found in the articles of Kulenović et al. [14], Kubiaczyk and Saker [13], and Greaf et al. [11].

In [18], infinite integral conditions for oscillation of all solutions of (1) in the case when *r*(*t*) ≡ 1 are obtained when the coefficient *p* takes some different ranges. A primary purpose of this paper is to further study the oscillation of solutions of (1). Our results extend and generalize some of the relevant results in [1–19].

Define the functions *z*(*t*) and *w*(*t*) as follows:

(5) z(t)=x(t)+px(t−τ),

(6) w(t)=z(t)+pz(t−τ).

If *x*(*t*) is an eventually positive solution of the equation

(7) $$\begin{array}{c} {\left(x\left(t\right)+px\left(t-\tau \right)\right)}^{\prime }+q\left(t\right)x\left(t-\sigma \right)=0, \end{array}$$

then *z*(*t*) and *w*(*t*) are also solutions of (7). Furthermore, *z*(*t*) is a differentiable solution, while *w*(*t*) is twice differentiable. (see Győri and Ladas [12]).

As usual, a solution of (1) is said to be oscillatory if it has arbitrarily large zeros and nonoscillatory if it is either eventually positive or eventually negative. Equation (1) is said to be oscillatory if all its solutions are oscillatory.

In the sequel, unless otherwise specified, when we write a functional inequality, we assume that it holds for all sufficiently large *t*.

## 2. Auxiliary Lemmas

To specify the proofs of our main results, we need the following essential lemmas.

**Lemma 1 (see [12]).**

Assume that (3) holds. Let *x*(*t*) be an eventually positive solution of (7). Then

(a) *z*(*t*) is a decreasing function and either
  (8) $$\begin{array}{c} \underset{t\to \infty }{\lim}\;z\left(t\right)=-\infty , \end{array}$$
  or
  (9) $$\begin{array}{c} \underset{t\to \infty }{\lim}\;z\left(t\right)=0. \end{array}$$
(b) The following statements are equivalent:
  (i) Equation (8) holds;
  (ii) *p* < −1;
  (iii) lim⁡_*t*→*∞*_ *x*(*t*) = *∞*;
  (iv) *w*(*t*) > 0, *w*′(*t*) > 0, *w*′′(*t*) > 0.
(c) The following statements are equivalent:
  (i) Equation (9) holds;
  (ii) *p* > −1;
  (iii) lim⁡_*t*→*∞*_ *x*(*t*) = 0;
  (iv) *w*(*t*) > 0, *w*′(*t*) < 0, *w*′′(*t*) > 0.

**Lemma 2 (see [16]).**

Assume that

(10) $$\begin{array}{c} \underset{t\to \infty }{\lim}\;\sup\overset{t+\tau_{i}}{\underset{t}{\int }}p_{i}\left(s\right)ds>0,\;\;for\;\;some\;\;i. \end{array}$$

If *x*(*t*) is an eventually positive solution of the delay differential equation

(11) $$\begin{array}{c} x^{\prime }\left(t\right)+\overset{n}{\underset{i=1}{\sum }}p_{i}\left(t\right)x\left(t-\tau_{i}\right)=0, \end{array}$$

then, for the same *i*,

(12) $$\begin{array}{c} \underset{t\to \infty }{\lim}\;\inf\;\frac{x\left(t-\tau_{i}\right)}{x\left(t\right)}<\infty . \end{array}$$

**Lemma 3 (see [16]).**

If the equation

(13) $$\begin{array}{c} x^{\prime }\left(t\right)+\overset{n}{\underset{i=1}{\sum }}\;p_{i}\left(t\right)x\left(t-\tau_{i}\right)=0 \end{array}$$

has an eventually positive solution, then one has eventually that

(14) $$\begin{array}{c} \overset{t+\tau_{i}}{\underset{t}{\int }}p_{i}\left(s\right)ds\leq 1;\;i=1,2,\ldots . \end{array}$$

**Lemma 4 (see [12]).**

Assume that

(15) $$\begin{array}{c} P_{i},\tau_{i}\in C\left[\left[t_{0},\infty \right),R^{+}\right],\;for\;i=1,2,\ldots ,n. \end{array}$$

Then the differential inequality

(16) $$\begin{array}{c} x^{\prime }\left(t\right)+\overset{n}{\underset{i=1}{\sum }}P_{i}\left(t\right)x\left(t-\tau_{i}\left(t\right)\right)\leq 0,\;t\geq t_{0} \end{array}$$

has an eventually positive solution if and only if the equation

(17) $$\begin{array}{c} x^{\prime }\left(t\right)+\overset{n}{\underset{i=1}{\sum }}P_{i}\left(t\right)x\left(t-\tau_{i}\left(t\right)\right)=0,\;t\geq t_{0} \end{array}$$

has an eventually positive solution.

## 3. Main Results

In this section, we establish some infinite integral conditions for all solutions of (1) to oscillate. We assume that condition (3) holds.

**Theorem 5 .**

Let conditions (2) and (3) hold with −1 ≤ *p* ≤ 0,

(18) $$\begin{array}{c} \overset{t+\sigma }{\underset{t}{\int }}\frac{q\left(s\right)}{r\left(s-\sigma \right)}ds>0, \end{array}$$

(19) $$\begin{array}{c} \overset{\infty }{\underset{t_{0}}{\int }}\left[\;\frac{q\left(t\right)}{r\left(t-\sigma \right)}\;\ln\left(e\overset{t+\sigma }{\underset{t}{\int }}\;\frac{q\left(s\right)}{r\left(s-\sigma \right)}ds\right)\right]dt=\infty .\; \end{array}$$

Then every solution of (1) is oscillatory.

**Proof**

Assume that (1) has a nonoscillatory solution on [*t*_0_, *∞*). Then, without loss of generality, there is a *t*_1_ ∈ [*t*_0_, *∞*), sufficiently large, so that *x*(*t*) > 0, *x*(*t* − *τ*) > 0 and *x*(*t* − *σ*) > 0 on [*t*_1_, *∞*). Set *z*(*t*) to be defined as in (5). Then by Lemma 1, it follows that

(20) $$\begin{array}{c} z\left(t\right)>0. \end{array}$$

As *x*(*t*) > *z*(*t*), it follows from (1) that

(21) $$\begin{array}{c} {\left(r\left(t\right)z\left(t\right)\right)}^{\prime }+q\left(t\right)z\left(t-\sigma \right)\leq 0. \end{array}$$

Dividing the last inequality by *r*(*t*) > 0, we obtain

(22) $$\begin{array}{c} z^{\prime }\left(t\right)+\frac{r^{\prime }\left(t\right)}{r\left(t\right)}z\left(t\right)+\frac{q\left(t\right)}{r\left(t\right)}\;z\left(t-\sigma \right)\leq 0. \end{array}$$

Let

(23) $$\begin{array}{c} z\left(t\right)=\exp\left(-\overset{t}{\underset{t_{0}}{\int }}\;\frac{r^{\prime }\left(s\right)}{r\left(s\right)}ds\right)y\left(t\right). \end{array}$$

This implies that *y*(*t*) > 0. Substituting in (22) yields

(24) $$\begin{array}{c} y^{\prime }\left(t\right)+\frac{q\left(t\right)}{r\left(t-\sigma \right)}y\left(t-\sigma \right)\leq 0,\;t\geq t_{0}. \end{array}$$

So by Lemma 4, we have that the delay differential equation

(25) $$\begin{array}{c} y^{\prime }\left(t\right)+\frac{q\left(t\right)}{r\left(t-\sigma \right)}y\left(t-\sigma \right)=0,\;t\geq t_{0} \end{array}$$

has an eventually positive solution as well. Let

(26) $$\begin{array}{c} \lambda \left(t\right)=-\frac{y^{\prime }\left(t\right)}{y\left(t\right)}. \end{array}$$

Then *λ*(*t*) is positive and continuous, and there exists *t*_1_ ≥ *t*_0_ such that *y*(*t*_1_) > 0, and

(27) $$\begin{array}{c} y\left(t\right)=y\left(t_{1}\right)\exp\left(-\overset{t}{\underset{t_{1}}{\int }}\lambda \left(s\right)ds\right). \end{array}$$

Furthermore, *λ*(*s*) satisfies the generalized characteristic equation

(28) $$\begin{array}{c} \lambda \left(t\right)=\overset{-}{Q}\left(t\right)\exp\left(\overset{t}{\underset{t-\sigma }{\int }}\lambda \left(s\right)ds\right), \end{array}$$

where

(29) $$\begin{array}{c} \overset{-}{Q}\left(t\right)=\frac{q\left(t\right)}{r\left(t-\sigma \right)}. \end{array}$$

Let

(30) $$\begin{array}{c} \Upsilon \left(t\right)=\overset{t+\sigma }{\underset{t}{\int }}\overset{-}{Q}\left(s\right)ds. \end{array}$$

Therefore

(31) $$\begin{array}{c} \lambda \left(t\right)=\overset{-}{Q}\left(t\right)\exp\left(\frac{1}{\Upsilon \left(t\right)}\Upsilon \left(t\right)\overset{t}{\underset{t-\sigma }{\int }}\lambda \left(s\right)ds\right). \end{array}$$

Applying the inequality (cf. Erbe et al. [8, page 32]),

(32) $$\begin{array}{c} e^{ax}\geq x+\frac{\ln\left(ea\right)}{a}\;\forall x,a>0, \end{array}$$

to (31), we have

(33) $$\begin{array}{c} \lambda \left(t\right)\geq \overset{-}{Q}\left(t\right)\left(\frac{1}{\Upsilon \left(t\right)}\overset{t}{\underset{t-\sigma }{\int }}\lambda \left(s\right)ds+\frac{\ln\left(e\Upsilon \left(t\right)\right)}{\Upsilon \left(t\right)}\right), \end{array}$$

or

(34) λ(t)(∫tt+σQ−(s)ds)−Q−(t)∫t−σtλ(s)ds  ≥Q−(t)(ln⁡ e∫tt+σQ−(s)ds).

Then, for *B* > *T*, we have

(35) ∫TBλ(t)(∫tt+σQ−(s)ds)dt−∫TBQ−(t)∫t−σtλ(s)ds dt  ≥∫TBQ−(t)(ln⁡ e∫tt+σ  Q−(s)ds)dt.  

By interchanging the order of integration, we get

(36) $$\begin{array}{c} \overset{B}{\underset{T}{\int }}\overset{-}{Q}\left(t\right)\left(\overset{t}{\underset{t-\sigma }{\int }}\lambda \left(s\right)ds\right)dt\geq \overset{B-\sigma }{\underset{T}{\int }}\left(\overset{s+\sigma }{\underset{s}{\int }}\overset{-}{Q}\left(t\right)\lambda \left(s\right)dt\right)ds. \end{array}$$

Hence

(37) $$\begin{array}{c} \overset{B}{\underset{T}{\int }}\overset{-}{Q}\left(t\right)\left(\overset{t}{\underset{t-\sigma }{\int }}\lambda \left(s\right)ds\right)dt\geq \overset{B-\sigma }{\underset{T}{\int }}\lambda \left(s\right)\left(\overset{s+\sigma }{\underset{s}{\int }}\overset{-}{Q}\left(t\right)dt\right)ds. \end{array}$$

Then

(38) $$\begin{array}{c} \overset{B}{\underset{T}{\int }}\overset{-}{Q}\left(t\right)\left(\overset{t}{\underset{t-\sigma }{\int }}\lambda \left(s\right)ds\right)dt\geq \overset{B-\sigma }{\underset{T}{\int }}\lambda \left(t\right)\left(\overset{t+\sigma }{\underset{t}{\int }}\overset{-}{Q}\left(s\right)ds\right)dt. \end{array}$$

From (35) and (38), we find that

(39) ∫B−σBλ(t)(∫tt+σQ−(s)ds)dt  ≥∫TBQ−(t)(ln⁡ e∫tt+σQ−(s)ds)dt.

However, using Lemma 3, it follows that

(40) $$\begin{array}{c} \overset{t+\sigma }{\underset{t}{\int }}\overset{-}{Q}\left(s\right)ds<1 \end{array}$$

eventually. Therefore, from (40) in (39), we get

(41) $$\begin{array}{c} \overset{B}{\underset{B-\sigma }{\int }}\lambda \left(t\right)dt\geq \overset{B}{\underset{T}{\int }}\overset{-}{Q}\left(t\right)\ln\left(e\overset{t+\sigma }{\underset{t}{\int }}\overset{-}{Q}\left(s\right)ds\right)dt. \end{array}$$

That is,

(42) $$\begin{array}{c} \frac{y\left(B-\sigma \right)}{y\left(B\right)}\geq \overset{B}{\underset{T}{\int }}\overset{-}{Q}\left(t\right)\ln\left(e\overset{t+\sigma }{\underset{t}{\int }}\overset{-}{Q}\left(s\right)ds\right)dt, \end{array}$$

which implies by condition (19) that

(43) $$\begin{array}{c} \underset{t\to \infty }{\lim}\frac{y\left(t-\sigma \right)}{y\left(t\right)}=\infty . \end{array}$$

On the other hand, from Lemma 2, we have

(44) $$\begin{array}{c} \underset{t\to \infty }{\lim}\frac{y\left(t-\sigma \right)}{y\left(t\right)}<\infty . \end{array}$$

This is a contradiction with (43). The proof is complete.

**Example 6 .**

Consider the equation

(45) [et+1(x(t)−12x(t−2))]′+et−1[1+tt]x(t−1)=0,    t≥e,

where

(46) r(t)=et+1,  q(t)=et−1[1+tt],p=−12, σ=1, τ=2.

Observe that

(47) $$\begin{array}{c} \frac{q\left(t\right)}{r\left(t-\sigma \right)}=\;\frac{e^{t-1}\left[\left(1+t\right)/t\right]}{e^{t+1-1}}=\frac{1}{e}\left(1+\frac{1}{t}\right). \end{array}$$

Then

(48) ∫tt+σq(s)r(s−σ)ds=1e∫tt+1(1+1s)ds>0,∫t0∞[q(t)r(t−σ)ln⁡(e∫tt+σq(s)r(s−σ)ds)]dt    =∫e∞[1e(1+1t)ln⁡(e∫tt+11e(1+1s)ds)]dt  ≥1e∫e∞ln⁡(1+ln⁡(1+1t))=  ∞.

All conditions of Theorem 5 are satisfied. Then all solutions of (45) oscillate.

**Theorem 7 .**

Let conditions (2) and (3) hold with −1 < *p*, *r*(*t*) ≡ *r* > 0, *σ* > *τ*. Assume further that *q*(*t*) is *τ*- periodic;

(49) $$\begin{array}{c} \frac{1}{r\left(1+p\right)}\overset{t+\sigma -\tau }{\underset{t}{\int }}q\left(s\right)ds>0; \end{array}$$

(50) $$\begin{array}{c} \overset{\infty }{\underset{t_{0}}{\int }}\left[\frac{q\left(t\right)}{r\left(1+p\right)}\ln\left(e\overset{t+\sigma -\tau }{\underset{t}{\int }}\frac{q\left(s\right)}{r\left(1+p\right)}ds\right)\right]dt=\infty .\; \end{array}$$

Then every solution of (1) is oscillatory.

**Proof**

Assume that (1) has a nonoscillatory solution on [*t*_0_, *∞*). Then, without loss of generality, there is a *t*_1_ ∈ [*t*_0_, *∞*), sufficiently large, so that *x*(*t*) > 0, *x*(*t* − *τ*) > 0 and *x*(*t* − *σ*) > 0 on [*t*_1_, *∞*). Let *z*(*t*) and *w*(*t*) be defined as in (5) and (6). It is easily seen, by direct substituting, that *z*(*t*) and *w*(*t*) are also solutions of (1) when *p* and *r* are constants; that is

(51) $$\begin{array}{c} rz^{\prime }\left(t\right)+prz^{\prime }\left(t-\tau \right)+q\left(t\right)z\left(t-\sigma \right)=0, \end{array}$$

(52) $$\begin{array}{c} rw^{\prime }\left(t\right)+prw^{\prime }\left(t-\tau \right)+q\left(t\right)w\left(t-\sigma \right)=0. \end{array}$$

By Lemma 1, we have that *z*(*t*) is decreasing and *w*(*t*) > 0. Also, we have indeed that

(53) w′(t)=−1rq(t)z(t−σ)≥−1rq(t)z(t−σ−τ)=−1r  q(t−τ)z(t−σ−τ)=w′(t−τ).

Then

(54) $$\begin{array}{c} w^{\prime }\left(t\right)\geq w^{\prime }\left(t-\tau \right). \end{array}$$

Using (54) in (52) implies that

(55) $$\begin{array}{c} r\left(1+p\right)w^{\prime }\left(t-\tau \right)+q\left(t\right)w\left(t-\sigma \right)\leq 0. \end{array}$$

As *p* > −1, we have 1 + *p* > 0. Then

(56) $$\begin{array}{c} w^{\prime }\left(t-\tau \right)+\frac{1}{r\left(1+p\right)}q\left(t\right)w\left(t-\sigma \right)\leq 0. \end{array}$$

In view of the *τ*-periodicity of *q*(*t*), (56) implies that

(57) $$\begin{array}{c} w^{\prime }\left(t\right)+\frac{1}{r\left(1+p\right)}q\left(t\right)w\left(t-\left(\sigma -\tau \right)\right)\leq 0. \end{array}$$

As *w*(*t*) is positive solution, so by Lemma 4, the delay differential equation

(58) $$\begin{array}{c} w^{\prime }\left(t\right)+\frac{1}{r\left(1+p\right)}q\left(t\right)w\left(t-\left(\sigma -\tau \right)\right)=0 \end{array}$$

has an eventually positive solution as well. Let

(59) $$\begin{array}{c} \lambda \left(t\right)=-\frac{y^{\prime }\left(t\right)}{y\left(t\right)}. \end{array}$$

Then *λ*(*t*) is positive and continuous, and there exists *t*_1_ ≥ *t*_0_ such that *y*(*t*_1_) > 0, and

(60) $$\begin{array}{c} y\left(t\right)=y\left(t_{1}\right)\exp\left(-\overset{t}{\underset{t_{1}}{\int }}\lambda \left(s\right)ds\right). \end{array}$$

Furthermore, *λ*(*s*) satisfies the generalized characteristic equation

(61) $$\begin{array}{c} \lambda \left(t\right)={\overset{-}{Q}}_{1}\left(t\right)\exp\left(\overset{t}{\underset{t-\sigma +\tau }{\int }}\lambda \left(s\right)ds\right), \end{array}$$

where

(62) $$\begin{array}{c} {\overset{-}{Q}}_{1}\left(t\right)=\frac{q\left(t\right)}{r\left(1+p\right)}. \end{array}$$

Let

(63) $$\begin{array}{c} \Upsilon_{1}\left(t\right)=\overset{t+\sigma -\tau }{\underset{t}{\int }}{\overset{-}{Q}}_{1}\left(s\right)ds. \end{array}$$

Therefore

(64) $$\begin{array}{c} \lambda \left(t\right)={\overset{-}{Q}}_{1}\left(t\right)\exp\left(\frac{1}{\Upsilon_{1}\left(t\right)}\Upsilon_{1}\left(t\right)\overset{t}{\underset{t-\sigma +\tau }{\int }}\lambda \left(s\right)ds\right). \end{array}$$

Applying the inequality (32) to (64), we have

(65) $$\begin{array}{c} \lambda \left(t\right)\geq {\overset{-}{Q}}_{1}\left(t\right)\left(\frac{1}{\Upsilon_{1}\left(t\right)}\overset{t}{\underset{t-\sigma +\tau }{\int }}\lambda \left(s\right)ds+\frac{\ln\left(e\Upsilon_{1}\left(t\right)\;\right)}{\Upsilon_{1}\left(t\right)}\right), \end{array}$$

or

(66)   λ(t)(∫tt+σ−τQ−1(s)ds)−Q−1(t)∫t−σ+τtλ(s)ds  ≥Q−1(t)(ln⁡ e∫tt+σ−τQ−1(s)ds).

Then, for *B* > *T*, we have

(67) ∫TBλ(t)(∫tt+σ−τQ−1(s)ds)dt−∫TBQ−1(t)∫t−σ+τtλ(s)ds dt  ≥  ∫TBQ−1(t)(ln⁡ e∫tt+σ−τQ−1(s)ds)dt.

By interchanging the order of integration, we get

(68) ∫TBQ−1(t)(∫t−σ+τtλ(s)ds)dt  ≥∫TB−σ+τ(∫ss+σ−τQ−1(t)λ(s)dt)ds.

Hence

(69) ∫TBQ−1(t)(∫t−σ+τtλ(s)ds)dt  ≥∫TB−σ+τλ(s)(∫ss+σ−τQ−1(t)dt)ds.

Then

(70) ∫TBQ−1(t)(∫t−σ+τtλ(s)ds)dt  ≥∫TB−σ+τλ(t)(∫tt+σ−τQ−1(s)ds)dt.

From (67) and (70), we find that

(71) ∫B−σ+τBλ(t)(∫tt+σ−τQ−1(s)ds)dt  ≥∫TBQ−1(t)(ln⁡ e∫tt+σ−τQ−1(s)ds)dt.  

However, using Lemma 3, it follows that

(72) $$\begin{array}{c} \overset{t+\sigma -\tau }{\underset{t}{\int }}{\overset{-}{Q}}_{1}\left(s\right)ds<1 \end{array}$$

eventually. Therefore, from (72) in (71), we get

(73) $$\begin{array}{c} \overset{B}{\underset{B-\sigma +\tau }{\int }}\lambda \left(t\right)dt\geq \overset{B}{\underset{T}{\int }}{\overset{-}{Q}}_{1}\left(t\right)\ln\left(e\overset{t+\sigma }{\underset{t}{\int }}{\overset{-}{Q}}_{1}\left(s\right)ds\right)dt \end{array}$$

or

(74) $$\begin{array}{c} \frac{y\left(B-\left(\sigma -\tau \right)\right)}{y\left(B\right)}\geq \overset{B}{\underset{T}{\int }}{\overset{-}{Q}}_{1}\left(t\right)\ln\left(e\overset{t+\sigma -\tau }{\underset{t}{\int }}{\overset{-}{Q}}_{1}\left(s\right)ds\right)dt, \end{array}$$

which implies by condition (50) that

(75) $$\begin{array}{c} \underset{t\to \infty }{\lim}\frac{y\left(t-\left(\sigma -\tau \right)\right)}{y\left(t\right)}=\infty . \end{array}$$

On the other hand, from Lemma 2, we have

(76) $$\begin{array}{c} \underset{t\to \infty }{\lim}\frac{y\left(t-\sigma \right)}{y\left(t\right)}<\infty . \end{array}$$

This is a contradiction with (75). The proof is complete.

**Example 8 .**

Consider the equation

(77) (x(t)−12x(t−π))′+(1+cos⁡2t)x(t−2π)=0,t>0,

where

(78) −1≤p=−12, σ=2π, τ=π,r(t)=1, q(t)=1+cos⁡2t.

Observe that

(79) 1r(1+p)∫tt+σ−τq(s)ds=11−1/2∫tt+π(1+cos⁡2s)ds=2[s+12sin2s|tt+π=2(t+π−t+sin2(t+π)−sin2t)=2(π+sin2t−sin2t)=2π>0.

Also,

(80) ∫t0∞[q(t)r(1+p)ln⁡(e∫tt+σ−τq(s)r(1+p)ds)]dt  =∫0∞2(1+cos⁡2t)ln⁡(e∫tt+π2(1+cos⁡2s)ds)dt  =∫0∞2(1+cos⁡2t)   ×[1+ln⁡(2∫tt+π(1+cos⁡2s)ds)]dt  =2(1+ln⁡2π)∫0∞(1+cos⁡2t)dt  =2(1+ln⁡2π)(t+12sin2t)|0∞=∞.

Then all conditions of Theorem 7 are satisfied and therefore all solutions of (77) oscillate.

**Remark 9 .**

Theorems 5 and 7 generalize and extend Theorems 3.1 and 3.2 of Saker and Elabbasy [18], respectively, and Theorem 6.4.3 in Gyori and Ladas [12], where *r*(*t*) ≡ 1. See also the results of Ahmed et al. [2] and Kubiaczyk and Saker [13].

## References

[1] Agarwal RP, Bohner M, Li W (2004). *Nonoscillation and Oscillation: Theory for Functional Differential Equations*.

[2] Ahmed FN, Ahmad RR, Din US, Noorani MS (2013). Oscillation criteria of first order neutral delay differential equations with variable coefficients. *Abstract and Applied Analysis*.

[3] Ahmed FN, Ahmad RR, Din UKS, Noorani MSM (2014). Some further results on oscillations for neutral delay differential equations with variable coefficients. *Abstract and Applied Analysis*.

[4] Al-Amri IR (2002). On the oscillation of first-order neutral delay differential equations with real coefficients. *International Journal of Mathematics and Mathematical Sciences*.

[5] Chuanxi Q, Ladas G (1990). Oscillations of first-order neutral equations with variable coefficients. *Monatshefte für Mathematik*.

[6] Dong J (2009). Oscillation of solutions for first order neutral differential equations with distributed deviating arguments. *Computers & Mathematics with Applications*.

[7] Elabbasy EM, Hassan TS, Saker SH (2005). Necessary and sufficient condition for oscillations of neutral differential equation. *Serdica Mathematical Journal*.

[8] Erbe LH, Kong Q, Zhang BG (1995). *Oscillation Theory for Functional-Differential Equations*.

[9] Gopalsamy K, Lalli BS, Zhang BG (1992). Oscillation of odd order neutral differential equations. *Czechoslovak Mathematical Journal*.

[10] Grammatikopoulos MK, Grove EA, Ladas G (1986). Oscillations of first-order neutral delay differential equations. *Journal of Mathematical Analysis and Applications*.

[11] Greaf JR, Savithri R, Thandapani E (2004). Oscillation of first order neutral delay differential equations. *Electronic Journal of Qualitative Differential Equations*.

[12] Győri I, Ladas G (1991). *Oscillation Theory of Delay Differential Equations*.

[13] Kubiaczyk I, Saker SH (2002). Oscillation of solutions to neutral delay differential equations. *Mathematica Slovaca*.

[14] Kulenović MRS, Ladas G, Meimaridou A (1987). Necessary and sufficient condition for oscillations of neutral differential equations. *Australian Mathematical Society Journal B: Applied Mathematics*.

[15] Ladas G, Sficas YG (1986). Oscillations of neutral delay differential equations. *Canadian Mathematical Bulletin*.

[16] Li B (1996). Oscillation of first order delay differential equations. *Proceedings of the American Mathematical Society*.

[17] Parhi N, Rath RN (2001). On oscillation and asymptotic behaviour of solutions of forced first order neutral differential equations. *Proceedings of the Indian Academy of Sciences: Mathematical Sciences*.

[18] Saker SH, Elabbasy EM (2001). Oscillation of first order neutral delay differential equations. *Kyungpook Mathematical Journal*.

[19] Yu JS, Wang Z, Qian CX (1992). Oscillation of neutral delay differential equations. *Bulletin of the Australian Mathematical Society*.

